# News and Events

**Published:** 2008

**Authors:** T. Ganesh

**Affiliations:** Department of Radiation Oncology, King Fahad Specialist Hospital, Dammam, Saudi Arabia

## 3D color x-ray system for material identification

Prof. Bob Cernik, working at the Diamond Light Source synchrotron facility in Oxfordshire, UK, has developed a prototype 3D color x-ray system to detect hidden explosives, drugs, or even cancer. Cernik's system uses "Tomographic Energy Dispersive Diffraction Imaging" (TEDDI). TEDDI measures voxels throughout a sample so that each contains an x-ray diffraction pattern - the key to identifying a material's atomic structure and chemistry. TEDDI requires an x-ray source (synchrotron) with a pencil-thin beam, a collimator, a detector, and much data analysis. The biggest application will be in the security industry, while TEDDI might also scan a biopsy sample in seconds to detect cancerous tissue, with the right programming.

[*From Guardian Unlimited dated March 20, 2008*]

**Recent publications of interest from International Atomic Energy Agency (IAEA)**

## Relative Biological Effectiveness in Ion Beam Therapy

Technical Reports Series No. 461

This publication covers all the aspects of the relative biological effectiveness (RBE) of ion beams, including laboratory measurements of RBE and the important variables that influence it, dose-related quantities and units, and approaches to the clinical use of the concept of RBE based on experimental findings, theoretical models, and previous clinical experience with fast neutrons and ions.

## Commissioning of Radiotherapy Treatment Planning Systems: Testing for Typical External Beam Treatment Techniques

IAEA TECDOC Series No. 1583

This publication is intended as a guide for the clinical commissioning of radiotherapy treatment planning systems (RTPSs) and provides a simple protocol for these tasks. The procedures for clinical commissioning tests cover typical treatment techniques used in the radiotherapy hospitals and are based on the use of a specific phantom. The purpose of this testing is twofold. Firstly, the tests will provide an educational opportunity for the user to become familiar with the operation of the RTPS. Secondly, the tests will demonstrate to the user that the logistic chain starting from CT scanning, anatomic modeling, treatment planning, and monitor unit (MU) calculation is operable and leads to the desired results with sufficient accuracy.

## Setting up a Radiotherapy Programme: Clinical, Medical Physics, Radiation Protection, and Safety Aspects

This publication provides guidance for designing and implementing radiotherapy programs, taking into account clinical, medical physics, radiation protection, and safety aspects. It reflects current requirements for radiotherapy infrastructure in settings with limited resources. It will be of use to professionals involved in the development, implementation, and management of radiotherapy programs.

[These publications can be freely downloaded from the IAEA website using the following links:

http://www-pub.iaea.org/MTCD/publications/PDF/trs461_web.pdf

http://www-pub.iaea.org/MTCD/publications/PDF/te_1583_web.pdf

http://www-pub.iaea.org/MTCD/publications/PDF/pub1296_web.pdf]

## National

1. **International Conference on Medical Physics-2008 and 29^th^ Annual Conference of Association of Medical Physicists of India**
  November 26-29, 2008, Mumbai, India Abstract Submission Deadline: July 31, 2008 For more details, log on to: www.icmp2008.com
2. **30^th^ Annual Conference of the Association of Radiation Oncologists of India**
  November 27-30, 2008, Mumbai, India Abstract Submission Deadline: August 30, 2008 For more details, log on to: www.aroicon2008.com
3. **International Conference on Radiation Biology and Translational Research in Radiation Oncology - in collaboration with 9^th^ Biennial Meeting of the Indian Society of Radiation Biology**
  November 10-12, 2008, Jaipur, India Abstract Submission Deadline: July 31, 2008 For more details, log on to: www.isrb-india.com, www.uniraj.ernet.in
4. **9th Asia Oceania Congress of Nuclear Medicine and Biology**
  Oct 31-Nov 4, 2008, New Delhi, India Abstract Submission Deadline: May 31, 2008 For more details, log on to: http://www.aofnmb2008.in/
5. **International Conference on Medical Physics, Radiation Protection and Radiobiology (ICMPRPR-2K9); and XIV Annual Convention of Northern Chapter of AMPI**
  February 11-13, 2009, Jaipur, India For more details: arunchougule11@gmail.com, arunchougle@rediffmail.com

## International

1. **50th Annual Meeting of the American Association of Physicists in Medicine**
  July 27-31, 2008, Houston, Texas, USA For more details, log on to: http://aapm.org/meetings/08AM/
2. **Asian-Pacific Congress of Medical Physics 2008, Taipei, Taiwan**
  Meeting Dates: June 21-22, 2008, Taipei, Taiwan For more details, log on to: http://www.nacmpa.org/taiwan2008/
3. **European Conference - Medical Physics and Engineering - 110 Years after the Discovery of Polonium and Radium**
  September 17-21, 2008, Krakow, Poland For more details, log on to: http://mpekrak08.ftj.agh.edu.pl/
4. **Engineering and Physical Sciences in Medicine and the Australian Biomedical Engineering Conference**
  November 16-20, 2008, Christchurch, New Zealand Abstract Submission Deadline: May 16, 2008 For more details, log on to: http://www.uco.canterbury.ac.nz/conference/epsm-abec/
5. **12th Congress of International Radiation Protection Association (IRPA 12): Strengthening Radiation Protection Worldwide**
  Nov 19-24, 2008, Buenos Aires, Argentina For more details, log on to: http://www.irpa12.org.ar/
6. **5th International Conference on Radiotherapy Gel Dosimetry**
  September 29-October 3, 2008, Hersonissos, Crete, Greece Abstract Submission Deadline: May 25, 2008 For more details, log on to: http://www.dosgel2008.gr/

